# Main Routes of Entry and Genomic Diversity of SARS-CoV-2, Uganda

**DOI:** 10.3201/eid2610.202575

**Published:** 2020-10

**Authors:** Daniel Lule Bugembe, John Kayiwa, My V.T. Phan, Phiona Tushabe, Stephen Balinandi, Beatrice Dhaala, Jonas Lexow, Henry Mwebesa, Jane Aceng, Henry Kyobe, Deogratius Ssemwanga, Julius Lutwama, Pontiano Kaleebu, Matthew Cotten

**Affiliations:** UK Medical Research Council–Uganda Virus Research Institute and London School of Hygiene and Tropical Medicine Uganda Research Unit, Entebbe, Uganda (D. Lule Bugembe, B. Dhaala, J. Lexow, D. Ssemwanga, P. Kaleebu, M. Cotten);; Uganda Virus Research Institute, Entebbe (J. Kiyawa, P. Tushabe, S. Balinandi, D. Ssemwanga, J. Lutwama, P. Kaleebu);; Erasmus Medical Center, Rotterdam, the Netherlands (M.V.T. Phan);; Uganda Ministry of Health, Kampala, Uganda (H. Mwebesa, J. Aceng, H. Kyobe);; UK Medical Research Council–University of Glasgow Centre for Virus Research, Glasgow, Scotland, UK (M. Cotten)

**Keywords:** COVID-19, 2019 novel coronavirus disease, coronavirus disease, SARS-CoV-2, severe acute respiratory syndrome coronavirus 2, viruses, respiratory infections, zoonoses, Uganda

## Abstract

We established rapid local viral sequencing to document the genomic diversity of severe acute respiratory syndrome coronavirus 2 entering Uganda. Virus lineages closely followed the travel origins of infected persons. Our sequence data provide an important baseline for tracking any further transmission of the virus throughout the country and region.

Severe acute respiratory syndrome coronavirus 2 (SARS-CoV-2) (*1*,*2*), the cause of coronavirus disease (COVID-19), has been spreading globally since it was first reported in Wuhan, China, on December 30, 2019 (*3*,*4*), infecting >10 million persons and causing massive disruption of daily lives and substantial economic consequences (*5*). Given the expanding pandemic and the absence of effective vaccines and antiviral drugs, the best strategy to control the spread of SARS-CoV-2 might be testing, contact tracing, and quarantining. Early implementation of diagnostic testing enables contact tracing and quarantining to reduce transmission in the community and can protect limited healthcare resources. 

The importation of SARS-CoV-2 into Africa was inevitable given the volume of air travel and movement of tourists, traders, and workers between countries. We document COVID-19 outbreak preparedness and response in Uganda, a landlocked country in East Africa with entry by international flight or overland from bordering countries. The experience in Uganda provides a unique opportunity to follow virus transmission when early strong interventions are applied. We describe the importation of COVID-19 into Uganda and SARS-CoV-2 genomic data acquired from local sequencing efforts.

## The Study

**Figure 1:**
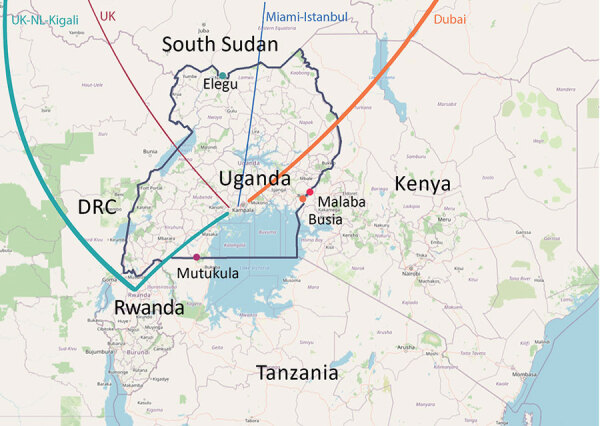
International flight routes of imported cases (colored lines) and the 4 main points of land entry into Uganda from Kenya, Tanzania, and South Sudan (colored dots).

Africa’s first case COVID-19 was recorded in Egypt on February 14, 2020 (*6*), and as of June 30, a total of 52 countries in Africa had reported cases. In anticipation of COVID-19 entry into Africa, the Uganda Virus Research Institute (UVRI) established SARS-CoV-2 diagnostics capacity in early February. The screening of all international arrivals and quarantine of suspected case-patients began March 19. The first COVID-19 case was detected in a returning traveler on March 21. Immediately after this first case was identified, a ban on international passenger flights was implemented on March 22, followed by a ban on local travel and public gatherings on March 27. After public health officials recognized that international truck drivers arriving with cargo from neighboring countries (primarily Kenya and Tanzania) posed a risk for virus importation, testing of truck drivers was initiated on April 13 at main border entry points (Figure 1) (https://www.health.go.ug/category/events-and-updates/page/4), and as of May 18, entry into Uganda required a negative SARS-CoV-2 test. A timeline shows various measures of public health preparedness and response, including testing activity, the total number of cases in Uganda, cases among truck drivers, and important intervention dates (Appendix Figure 1).

As of June 30, public health officials in Uganda had detected >1,500 cases in the country or at points of entry and had conducted >150,000 diagnostics tests. Approximately 2,000 tests per day have been performed at UVRI, which is designated as a Center of Excellence for Evaluation of COVID-19 Diagnostics by the Africa Centres for Disease Control and Prevention, by using real-time reverse transcription PCR assays on respiratory swabs samples from suspected case-patients (*7*). To facilitate virus tracing, we established local sequencing capacity to determine full viral genome sequences from confirmed COVID-19 case-patients. 

We report 20 SARS-CoV-2 genomic sequences from Uganda, obtained from 14 persons arriving from regions with circulating SARS-CoV-2 and 6 truck drivers screened at Uganda points-of-entry (Table; Figure 1). This study was approved by the UVRI Research and Ethics Committee (approval no. 00001354, study reference no. GC/127/20/04/771). 

**Table:** Summary characteristics of SARS-CoV-2 genomes obtained from 20 persons entering Uganda*

Genome	GISAID ID†	Sample date	C_t_	Patient age, y	Patient travel history	Lineage‡
hCoV-19/Uganda/UG001/2020	EPI_ISL_451183	2020 Mar 23	19	48	Miami to Istanbul	A
hCoV-19/Uganda/UG002/2020	EPI_ISL_451184	2020 Mar 26	19	43	Dubai	A
hCoV-19/Uganda/UG003/2020	EPI_ISL_451185	2020 Mar 27	22	10	UK	B.1.1
hCoV-19/Uganda/UG004/2020	EPI_ISL_451186	2020 Mar 27	18	25	UK to NL to Rwanda	B.1.1.1
hCoV-19/Uganda/UG005/2020	EPI_ISL_451187	2020 Mar 27	18	26	UK to NL to Rwanda	B
hCoV-19/Uganda/UG006/2020	EPI_ISL_451188	2020 Mar 30	23	27	UK to NL to Rwanda	B
hCoV-19/Uganda/UG007/2020	EPI_ISL_451189	2020 Mar 30	21	8	UK to NL to Rwanda	B.1.1.1
hCoV-19/Uganda/UG008/2020	EPI_ISL_451190	2020 Mar 30	22	7	UK to NL to Rwanda	B.1.1.1
hCoV-19/Uganda/UG009/2020	EPI_ISL_451191	2020 Mar 30	20	9	UK to NL to Rwanda	B.1.1.1
hCoV-19/Uganda/UG010/2020	EPI_ISL_451192	2020 Mar 30	22	27	UK to NL to Rwanda	B.1.1.1
hCoV-19/Uganda/UG011/2020	EPI_ISL_451193	2020 Mar 30	21	29	Contact	B.4
hCoV-19/Uganda/UG012/2020	EPI_ISL_451194	2020 Mar 22	24	37	Dubai	A
hCoV-19/Uganda/UG013/2020	EPI_ISL_451195	2020 Mar 22	23	35	Dubai	B
hCoV-19/Uganda/UG014/2020	EPI_ISL_451196	2020 Mar 25	27	31	Dubai	B.1.1.1
hCoV-19/Uganda/UG015/2020	EPI_ISL_451197	2020 Apr 27	16	27	Kenya, by truck	B.1
hCoV-19/Uganda/UG016/2020	EPI_ISL_451198	2020 Apr 27	19	52	Kenya, by truck	B.1
hCoV-19/Uganda/UG017/2020	EPI_ISL_451199	2020 Apr 20	22	42	Tanzania, by truck	A
hCoV-19/Uganda/UG018/2020	EPI_ISL_451200	2020 May 1	28	22	Tanzania, by truck	B.1
hCoV-19/Uganda/UG019/2020	EPI_ISL_451201	2020 Apr 30	29	39	Kenya, by truck	B.1
hCoV-19/Uganda/UG020/2020	EPI_ISL_451202	2020 May 1	25	47	Kenya, by truck	B.1

*C_t_, cycle threshold (based on diagnostic real-time reverse transcription PCR; NL, the Netherlands; SARS-CoV-2, severe acute respiratory syndrome coronavirus 2; UK, United Kingdom. †Virus genomes sequences available from GISAID (https://www.gisaid.org). ‡SARS-CoV-2 lineages determined by using CoV-GLUE (*13*).

**Figure 2:**
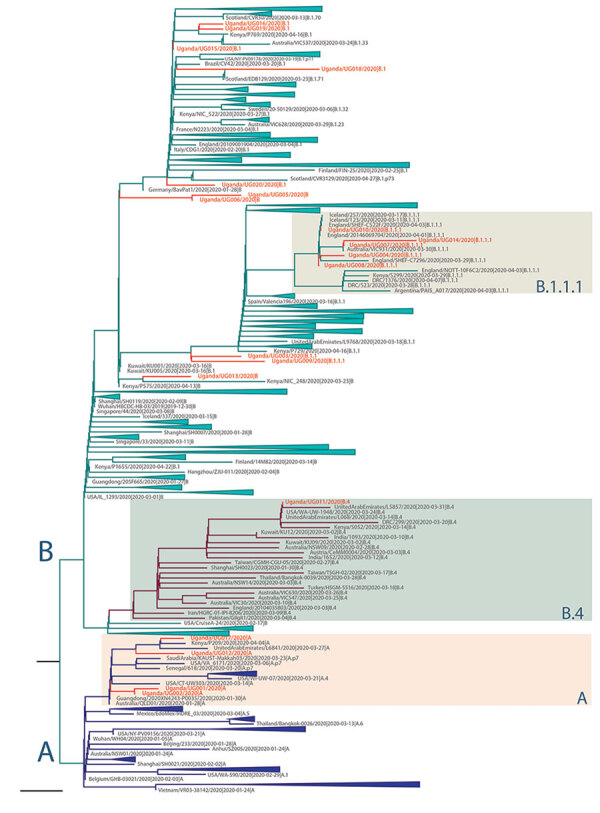
Maximum-likelihood phylogenetic tree of severe acute respiratory syndrome coronavirus 2 (SARS-CoV-2) genomes in Uganda. The full SARS-CoV-2 genomes used for phylogenetic lineage nomenclature (A. Rambaut et al., unpub. data, https://doi.org/10.1101/2020.04.17.046086) as defined on May 19, 2020, were retrieved from GISAID (http://www.gisaid.org) (*8*). Identical sequences were removed, and a total of 395 global representative sequences from each phylogenetic lineage type were selected for further phylogenetic analyses. The reported Uganda sequences, combined with the global SARS-CoV-2 sequences, were aligned by using MAFFT (*9*) and untranslated regions at 5′ and 3′ were trimmed. Maximum-likelihood phylogenetic tree was constructed in RAxML (*10*), under the general time-reversible plus gamma distribution model as best-fitted substitution model determined by IQ-TREE (*11*) and run for 100 pseudo-replicates. The resulting tree was visualized in Figtree (*12*) and rooted at the point of splitting lineage A and B. Scale bar indicates 6 × 10^–5^ nucleotide substitutions per site. The branch length is drawn to the scale of nucleotide substitutions per site. The Uganda genomes are indicated in red. The 2 major lineages of SARS-CoV-2 (A and B) are indicated to the left of the tree; the main groups of the Uganda genomes (A, B1.1.1, B4) are indicated by colored boxes to the right of the tree.

We compared the 20 SARS-CoV-2 genomes detected in Uganda with genomes detected globally. The Uganda genomes belonged to phylogenetic lineages A, B, B.1, B.1.1, B.1.1.1, and B.4, among which lineage B.1 has the largest number of sequences that have spread to >20 countries in Europe, the Americas, Asia, and Australia (https://github.com/hCoV-2019/lineages). Genome UG001 (from a traveler arriving from the United States), genomes UG002 and UG012 (from travelers arriving from Dubai), and genome UG017 (from a truck driver from Tanzania) fall within SARS-CoV-2 lineage A (A. Rambaut et al., unpub. data, https://doi.org/10.1101/2020.04.17.046086), with the nearest known genomes occurring in Asia, Australia, Kenya, and the United States (Figure 2). Genome UG011 was from a contact of a Uganda case-patient and is most related to USA/WA-UW-1948 and UnitedArabEmirates/L068 strains within lineage B.4 (Figure 2). Genomes UG004, UG007, UG008, and UG010 were detected in a group of travelers returning from the United Kingdom; these genomes fall within lineage B.1.1.1, which included other United Kingdom–derived genomes (Figure 2). Also in this lineage is genome UG014, detected in a traveler returning from Dubai. Additional sequences from a traveling group (UG005 and UG006) were assigned to lineage B, whereas UG003 (assigned to lineage B.1.1) and UG009 (assigned to lineage B.1.1.1) were closely related to the lineage B.1.1.1, containing genomes from the traveling group in whom genomes UG004, UG007, UG008, and UG010 were detected. Genome UG013 (from a traveler returning from Dubai) belonged to lineage B and was closely related to strains from Asia and Kenya. SARS-CoV-2 genomes identified from returning travelers from Dubai belonged to different lineages (UG002 and UG012 of lineage A, UG013 of lineage B, and UG014 of lineage B.1.1.1), suggesting these travelers contracted the virus from multiple sources despite sharing similar travel routes. 

In addition to air traffic, another means of SARS-CoV-2 entry into Uganda is with drivers of cargo trucks entering the country through 4 main entry points from Kenya, Tanzania, and South Sudan (Figure 1). All 4 genomes from truck drivers from Kenya belonged to lineage B.1, whereas genomes from truck drivers from Tanzania belonged to lineage A and B.1 (Table). The truck driver viral genomes did not cluster closely with any current local Uganda genomes, suggesting that these truck drivers contracted the virus outside Uganda, although the sample size is too small for firm conclusions. Careful monitoring and additional sequence data from truck driver and community cases will enable an estimate of the amount of transmission that might occur between truck drivers and the general population of Uganda.

An indication of the current SARS-CoV-2 genomic sequence diversity (Appendix Figure 2) is the single nucleotide changes from the original Wuhan-1 strain (GenBank accession no. NC_045512). The Uganda strains differ at 5–20 positions across the ≈30 kb genome, including a small number of changes in the spike protein–coding region, which is a main target for vaccines. The spike protein showed 1 polymorphism with the lineage A viruses (including 4 Uganda virus sequences), encoding D614, whereas all other clades encoded G614 in the spike protein.

## Conclusions

We describe the initial SARS-CoV-2 genomes imported into Uganda. We observed 6 lineages among 20 genomes, which were imported through returning air travelers and truck drivers entering Uganda. We shared all sequences with the public health community by depositing in the GISAID public database (https://www.gisaid.org, accession nos. EPI_ISL_451183–202) (*8*).

Since the governmental ban on international flights was implemented in the last week of March, no further imported COVID-19 cases from international air travelers into Uganda have been reported, underscoring the effectiveness of these policy measures. However, the increasing detection of SARS-CoV-2 in apparently healthy truck drivers is concerning. The quantity of viral RNA levels in some truck driver samples is high (cycle threshold values 16–19), yet these persons were still capable of driving a truck, indicating mild symptoms. This combination of high viral levels and sufficient health to continue normal activities could lead to further spread of the virus within the community without effective quarantine measures. The current efforts to increase community testing and truck drivers contact tracing and quarantine are essential to identify new cases and prevent further spread of the virus in Uganda.

Appendix Additional information about main routes of entry and genomic diversity of SARS-CoV-2, Uganda.
