# Supplementary material for: Main Routes of Entry and Genomic Diversity of SARS-CoV-2, Uganda
Source: Emerg Infect Dis. 2020 Oct;26(10):2411–5. doi: 10.3201/eid2610.202575 (PMC7510740; doi:10.3201/eid2610.202575)
Supplement: Appendix — Additional information about main routes of entry and genomic diversity of SARS-CoV-2, Uganda. [file 20-2575-Techapp-s1.pdf]

Article DOI: https://doi.org/10.3201/eid2610.202575

Main Routes of Entry and Genomic Diversity of SARS-CoV-2, Uganda

Appendix

Diagnostics and Whole-Genome Sequencing of SARS-CoV-2

Total nucleic acid was extracted from respiratory swabs using Qiagen extraction kits and was subjected to quantitative RT-PCR using established diagnostic primers (*1*). From confirmed positive samples, residual nucleic acid from the PCR testing was converted to cDNA and SARS-CoV-2 sequences were amplified with PCR and amplicons spanning the entire genome using a modification of the ARTIC Network protocol (*2*). The resulting DNA amplicons were processed into sequencing libraries, barcoded separately and pooled to sequence on MinION R.9.4.1 flowcells. Reads were basecalled and demultiplexed from fast5 files using Guppy 3.5 or 3.6 (*3*) running on the UMIC HPC. Resulting reads were processed to remove chimeric reads and adapters as previously described (*4*) and primers were removed using porechop (*5*). Reads were then mapped to the reference genome Wuhan-1 strain (GenBank NC_045512) using minimap2 (*6*) and consensus genomes were generated in Geneious (Biomatters Ltd), polished using Medaka (*7*) and SNPs and mismatches were checked and resolved by consulting short reads.

References

1. Corman VM, Landt O, Kaiser M, Molenkamp R, Meijer A, Chu DK, et al. Detection of 2019 novel coronavirus (2019-nCoV) by real-time RT-PCR. Euro Surveill. 2020;25. PubMed https://doi.org/10.2807/1560-7917.ES.2020.25.3.2000045

2. Quick J. nCoV-2019 sequencing protocol [cited 2020 May 7]. https://www.protocols.io/view/ncov-2019-sequencing-protocol-bbmuik6w

3. Oxford Nanopore Technologies. Guppy, local accelerated basecalling for Nanopore data [cited 2020 May 7]. https://community.nanoporetech.com/downloads

4. Arias A, Watson SJ, Asogun D, Tobin EA, Lu J, Phan MVT, et al. Rapid outbreak sequencing of Ebola virus in Sierra Leone identifies transmission chains linked to sporadic cases. Virus Evol. 2016;2:vew016. PubMed https://doi.org/10.1093/ve/vew016

5. Wick R. Porechop [cited 2020 May 7]. https://github.com/rrwick/Porechop

6. Li H. Minimap2: pairwise alignment for nucleotide sequences. Bioinformatics. 2018;34:3094–100. PubMed https://doi.org/10.1093/bioinformatics/bty191

7. Oxford Nanpore Technologies. Medaka v1.0.1 [cited 2020 May 7]. https://github.com/nanoporetech/medaka/releases

8. Katoh K, Standley DM. MAFFT multiple sequence alignment software version 7: improvements in performance and usability. Mol Biol Evol. 2013;30:772–80. PubMed https://doi.org/10.1093/molbev/mst010

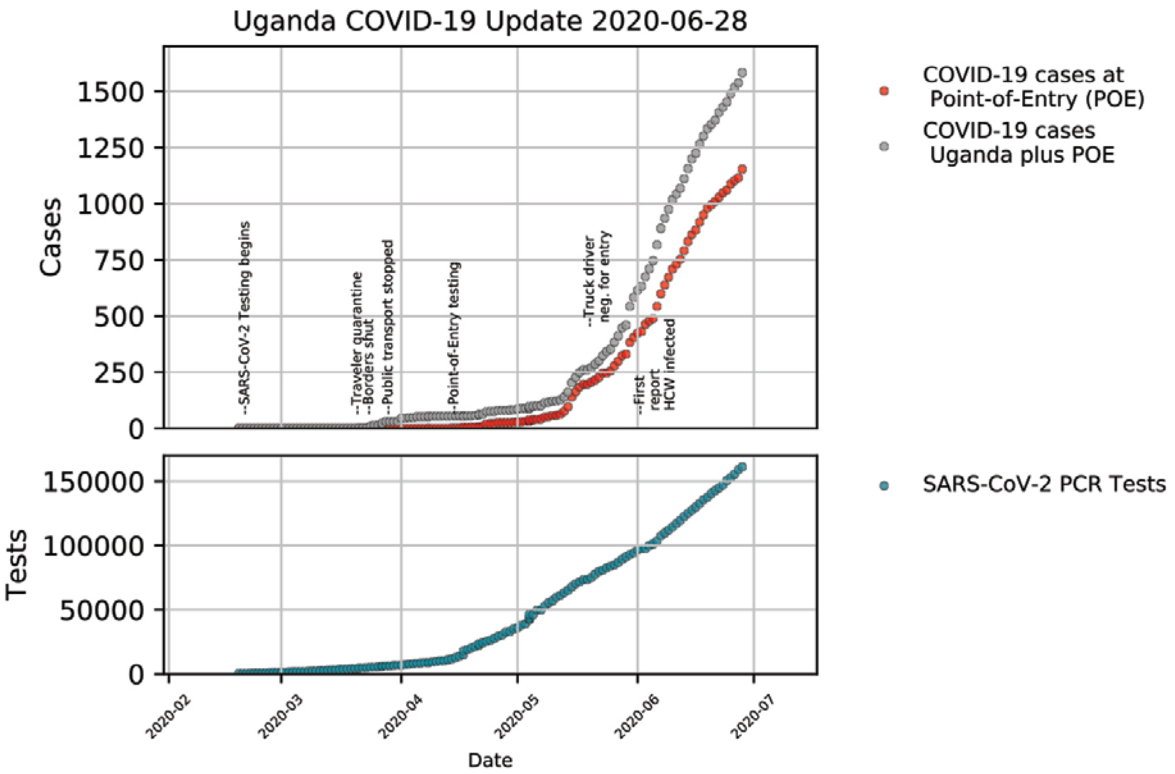

Appendix Figure 1. Timeline of COVID-19 epidemic in Uganda. Cumulative SARS-CoV-2 PCR-positive persons detected in Uganda and at points of entry (gray circles, upper panel), and those at points of entry only (red circles, upper panel), total performed SARS-CoV-2 PCR tests (green circles, lower panel), and major dates of COVID-19 interventions are indicated in text. Recently, Uganda has been returning infected non-Ugandan SARS-CoV-2 PCR-positive persons to their home countries, thus reducing the number of cases within the country. The numbers reported here are for all detected cases (in Uganda plus those imported at points of entry), rather than only cases in Uganda. Testing data were obtained from the Twitter accounts of the Uganda Ministry of Health (@MinofHealthUG) and the Uganda Virus Research Institute (@UVRIug).

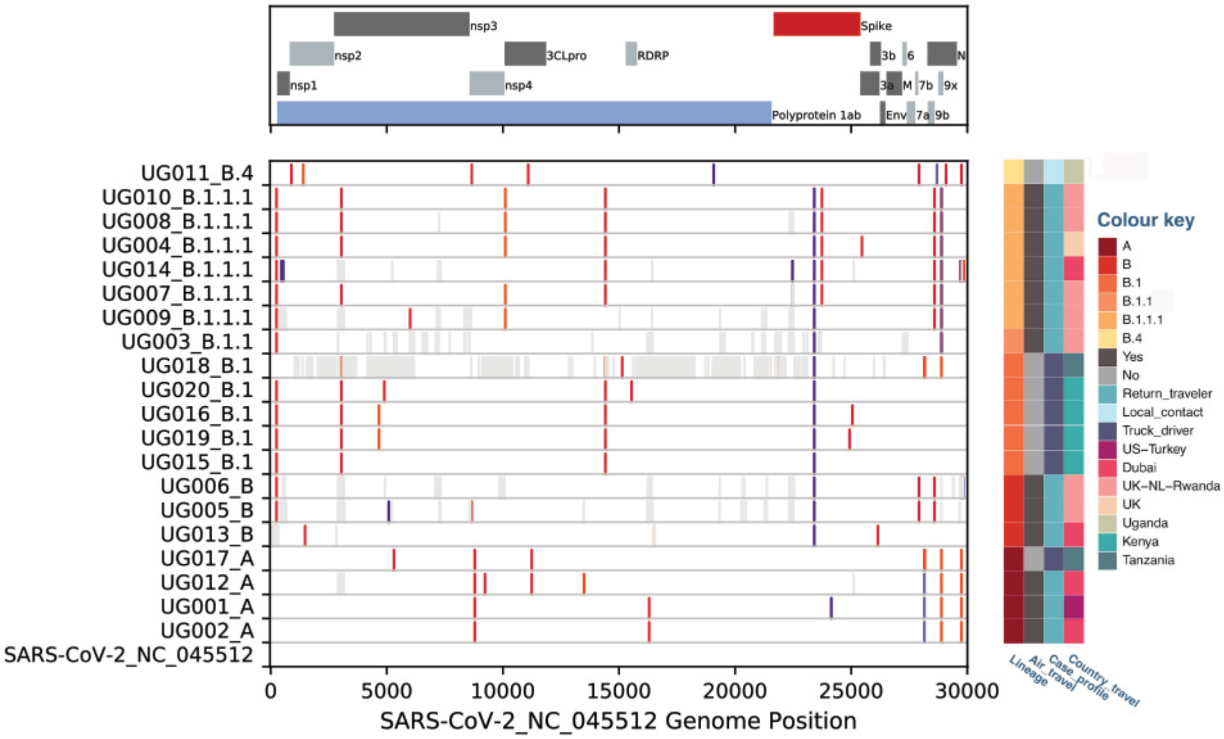

Appendix Figure 2. Nucleotide differences between Uganda SARS-CoV-2 genomes and the Wuhan-1 SARS-CoV-2 reference strain. Genome sequences were aligned in MAFFT (*8*). Nucleotide differences from the SARS-CoV-2 reference strain (GenBank accession no. NC_045512) were determined by using a python script. Changes are indicated by colored bars (change to A, orange; change to T, red; change to G, dark blue; change to C; light blue; gap, gray). Viral lineages, travel type, case profile, and country of travel are show to the right of the panel. The open reading frames of the SARS-CoV-2 genome are shown in the upper panel.
